# The impact of physical exercise on sleep quality among college students: a moderated mediation model

**DOI:** 10.3389/fpsyt.2026.1884052

**Published:** 2026-07-29

**Authors:** Xiaoyan Li, Wei Gao, Zhiheng Li, Ji Zhu, Yanhao Wang, Ming Li

**Affiliations:** School of Physical Education and Sports Science, Fujian Normal University, Fuzhou, Fujian, China

**Keywords:** college students, mobile phone addiction tendency, moderated mediation, physical exercise, resilience, sleep quality

## Abstract

**Objective:**

To explore the relationship between physical exercise and sleep quality among college students, and to examine the mediating role of mobile phone addiction tendency and the moderating role of psychological resilience, in order to reveal the underlying pathway linking physical exercise and sleep quality.

**Methods:**

A sample of 1,583 college students from Fuzhou, Fujian Province, was recruited and assessed using the Physical Activity Rating Scale-3 (PARS-3), the Pittsburgh Sleep Quality Index (PSQI), the Mobile Phone Addiction Tendency Scale for College Students, and the Connor-Davidson Resilience Scale (CD-RISC). The moderated mediation model was tested using SPSS 27.0 and the PROCESS macro Models 4 and 7.

**Results:**

(1) Physical exercise was significantly negatively associated with sleep quality (*β* = −0.19, *p* < 0.001). (2) Mobile phone addiction tendency mediated the relationship between physical exercise and sleep quality, with an indirect effect of −0.03, 95% CI [−0.04, −0.02], accounting for 13.64% of the total effect. (3) Psychological resilience significantly moderated the relationship between physical exercise and mobile phone addiction tendency (*β* = −0.31, 95% CI [−0.35, −0.27], *p* < 0.001; index of moderated mediation = −0.10, 95% CI [−0.12, −0.09]). Simple slope analysis revealed a crossover interaction pattern: physical exercise was positively associated with mobile phone addiction tendency among students with low psychological resilience (*β* = 0.18, 95% CI [0.13, 0.23], *p* < 0.001), but negatively associated with mobile phone addiction tendency among those with high psychological resilience (*β* = −0.36, 95% CI [−0.41, −0.31], *p* < 0.001).

**Conclusion:**

Physical exercise is significantly associated with better sleep quality among college students, both directly and indirectly through its association with lower mobile phone addiction tendency. Psychological resilience moderates the direction of the first stage of this indirect pathway, such that physical exercise is associated with reduced mobile phone addiction tendency only among individuals with moderate to high psychological resilience. These findings provide empirical evidence for developing targeted, resilience-informed exercise-based intervention strategies to promote sleep health among college students.

## 1 Introduction

Sleep disturbances are highly prevalent among college students worldwide and can seriously affect their health and quality of life (1). Currently, college students commonly exhibit irregular daily routines and excessive screen time before bed, and the number of those suffering from sleep disorders is increasing; 62% of college students report poor sleep quality (2). Furthermore, contemporary college students face pressures from academic study, daily life, and employment, and the quality of their sleep directly influences their physical and mental health as well as their learning and work efficiency (3). Research has shown that chronic sleep deprivation leads to cognitive decline, including inattention, memory impairment, and weakened logical thinking, which directly affects students’ academic performance and achievement (4). More seriously, sleep problems can trigger a range of psychological issues, such as elevated anxiety and depression, and even increase the risk of cardiovascular and metabolic diseases (5–8). The sleep habits formed during college often exert a lasting influence on individuals’ long-term health and development; unhealthy sleep patterns established in college may persist into the working stage, resulting in enduring health problems. In addition, sleep problems can create a vicious cycle with behaviors such as mobile phone dependence and unhealthy eating, and may exacerbate individuals’ stress perception (9). It is evident that sleep problems have become one of the major public health issues affecting college students’ physical health.

A large body of research indicates that physical exercise is a key factor in improving sleep quality among college students. Regular long-term physical exercise, especially aerobic and mind-body exercises, is associated with better subjective sleep quality, prolong sleep duration, and increase sleep efficiency (10). Additionally, with the increasing proliferation of the Internet, mobile phone addiction tendency has drawn growing scholarly attention. Previous studies have demonstrated that smartphone addiction is positively associated with sleep problems and depressive symptoms, and that poor sleep quality mediates the association between smartphone addiction and depressive symptoms among college students (11). Moreover, psychological resilience has often been examined as a moderator or mediator. Research has confirmed that psychological resilience moderates the relationship between sleep reactivity and sleep quality, with individuals higher in resilience exhibiting better sleep quality (12). Studies have also shown that psychological resilience reduces the direct associations among parental psychological control, psychological reactance, and problematic smartphone use, thereby attenuating their interrelationships. Psychological resilience plays a critical role in protecting adolescents from the adverse effects of parental psychological control on problematic smartphone use (13). Although the positive relationship between physical exercise and sleep quality has been confirmed by multiple studies, the underlying processes involving the mediating role of mobile phone addiction tendency and the moderating role of psychological resilience remain unclear. Therefore, the present study constructed a moderated mediation model to reveal the intrinsic pathway linking physical exercise and sleep quality.

Bandura’s social cognitive theory explains human behavior and functioning from the perspective of triadic reciprocal determinism, in which cognitive factors, personal factors, behaviors, and environmental events all influence one another (14). The core variable in this motivational theory is self-efficacy, defined as an individual’s confidence in their ability to successfully execute the actions necessary to meet situational demands (15). Self-efficacy serves as the driving force of psychological resilience; the higher the self-efficacy, the greater the psychological resilience. Engaging in regular physical exercise on a weekly basis can effectively enhance college students’ self-efficacy, thereby strengthening their psychological resilience. Bandura’s social cognitive theory particularly emphasizes the central role of cognitive factors in behavior change, providing a solid theoretical foundation for the present study to explore the psychological pathways underlying physical exercise.

### 1.1 Physical exercise and sleep quality

Physical exercise refers to planned, structured, repetitive bodily movement with the ultimate or intermediate goal of improving or maintaining physical fitness (16). Poor sleep quality has been demonstrated to be associated with a range of adverse mental health outcomes, such as depression (17), anxiety (18), and psychotic symptoms (19), and can inhibit the development of positive psychological qualities, such as resilience (20). Research has found that after long-term regular physical exercise, exercisers can experience significant improvements in negative emotions such as anxiety and depression, thereby enhancing sleep quality (21). A study by Wang & Bíró (22) further indicated that physical activity and healthy social relationships contribute to better sleep, whereas caffeine intake, stress, and irregular sleep–wake patterns reduce sleep quality. Moreover, a meta-analysis demonstrated that physical exercise significantly improves sleep quality and depressive symptoms in both adults and older adults, with aerobic exercise yielding the greatest effect (23). The above studies have consistently found that physical exercise is associated with sleep quality in college students. Based on this, we propose the following hypothesis:

H 1: Physical exercise may be positively associated with sleep quality among Chinese college students.

### 1.2 The mediating role of mobile phone addiction tendency

Mobile phone addiction tendency, also referred to as problematic mobile phone use or mobile phone dependency, is an important form of behavioral addiction tendency. It refers to a state in which individuals develop psychological dependence due to excessive smartphone use, subsequently losing self-control over their smartphone use, which impairs their social functioning and leads to psychological or behavioral problems (24, 25), with symptoms such as tolerance, withdrawal, salience, mood modification, craving, and loss of control (26). This behavior is characterized by several core features: an inability to control mobile phone use, constantly attending to phone notifications while neglecting the present scene and real-life environment (27, 28); the emergence of withdrawal symptoms, such as anxiety, irritability, anger, and physical discomfort like restlessness when unable to use the phone (25); and impairment in physical functioning, with excessive phone use significantly affecting academic performance and work efficiency, damaging real-life interpersonal interactions, and even causing health problems such as circadian rhythm disruption and vision loss (29). As an unhealthy behavior, mobile phone addiction tendency is highly detrimental to sleep. Existing research has demonstrated that the more severe college students’ mobile phone addiction tendency is, the poorer their sleep quality (30). Furthermore, a review incorporating 40 articles also found that college students with mobile phone addiction tendency are more likely to experience high levels of anxiety, depression, and impulsive behaviors, accompanied by poor sleep quality (31).

Both physical exercise and smartphone use behaviors are important factors influencing college students’ sleep quality. Research has shown that exercise interventions may have positive effects on ameliorating smartphone addiction tendency, suggesting that prolonged intervention duration may yield stronger intervention effects. Moreover, individuals with severe addiction benefit more from exercise participation compared to those with mild-to-moderate addiction tendency levels (32). In addition, experimental research has found that 30 minutes of acute aerobic exercise can improve inhibitory control functions in college students with smartphone addiction tendency, thereby reducing their level of mobile phone addiction tendency, which is of great significance for fostering healthy behavioral habits among college students (33). Notably, nighttime exercise can effectively reduce sleep delays caused by problematic smartphone use before bedtime. Among these nighttime exercisers, the frequency and duration of nighttime exercise are significantly associated with a lower probability of smartphone use before bedtime. Furthermore, the frequency and duration of nighttime exercise are correlated with lower levels of smartphone addiction tendency and anxiety disorders (34). Therefore, the present study proposes the following hypothesis:

H 2: Mobile phone addiction tendency may play a mediating role in the relationship between physical exercise and sleep quality.

### 1.3 The moderating role of psychological resilience

Definitions of psychological resilience can be broadly classified into trait-based, outcome-based, and process-based conceptualizations. The trait theory views psychological resilience as a relatively stable personality trait. The process theory emphasizes that psychological resilience is a dynamic process of interaction between the individual and the environment. The outcome theory focuses on defining psychological resilience in terms of developmental outcomes. The trait-based definition proposed by Werner (35) describes psychological resilience as the capacity of an individual to withstand high levels of destructive change while exhibiting as few maladaptive behaviors as possible. The outcome-based definition advanced by Masten (36) considers resilience as a class of phenomena characterized by positive adaptation and development despite serious threats. The process-based definition put forward by Tusaie & Dyer (37) refers to psychological resilience as a process through which a constellation of capacities and characteristics dynamically interact to enable individuals to recover rapidly and cope successfully in the face of significant stress and adversity. Thus, the trait-based, outcome-based, and process-based definitions each elucidate the essence of psychological resilience from different perspectives.

In recent years, a growing body of research has found that psychological resilience is closely related to psychological problems such as depression, stress, health, and anxiety. For example, Borrega-Mouquinho et al. (38) demonstrated that high-intensity interval training and moderate-intensity training significantly reduced stress, anxiety, and depression and enhanced resilience. Individuals with higher levels of resilience are generally emotionally stable, possess strong social adaptability and emotion regulation skills, are better able to focus on goals when facing tasks, cope with negative life and learning events with a positive attitude, and refrain from academic procrastination (39). Furthermore, previous research has found that psychological resilience exerts an inhibitory effect on mobile phone addiction tendency, and thus increasing psychological resilience can amplify the role of physical activity (40). There is a significant positive correlation between smartphone addiction and both anxiety and depression, and psychological resilience mediates these relationships; research suggests that higher levels of resilience can mitigate the adverse psychological effects of smartphone addiction (41). Therefore, as an important positive factor, psychological resilience can enhance the positive effects of physical exercise. In other words, the association between physical exercise and mobile phone addiction tendency may be moderated by psychological resilience. Based on the above research, we propose the following hypothesis:

H 3: Psychological resilience may play a moderating role in the relationship between physical exercise and mobile phone addiction tendency.

### 1.4 Research objective

**Figure 1:**
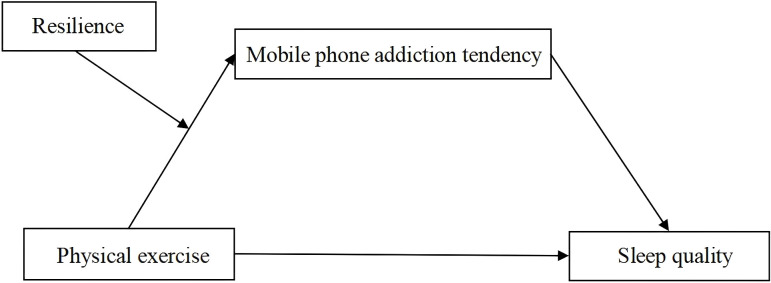
The proposed moderated mediation model.

Although the specific pathways have yet to be fully elucidated, a large body of evidence suggests that physical exercise, sleep quality, mobile phone addiction tendency, and psychological resilience are significantly interrelated. We propose integrating these factors into a comprehensive model to provide references and support for improving college students’ sleep quality. Accordingly, the present study constructed a moderated mediation model (**Figure 1**).

This study aims to investigate the effect of physical exercise on college students’ sleep quality, and to analyze the mediating role of mobile phone addiction tendency and the moderating role of psychological resilience in this relationship. The findings intend to reveal the associations among these variables and, on that basis, offer corresponding recommendations to provide references and support for enhancing sleep quality among Chinese college students.

## 2 Methods

### 2.1 Participants

The target sample size was determined based on recommendations for regression-based mediation and moderation analyses. Green (42)Fritz & Mackinnon (43) demonstrated that a minimum of 124 participants is required to detect a medium effect size (*f²* = 0.15) with 80% power at *α* = 0.05 for regression models with up to 10 predictors. However, to ensure adequate statistical power for detecting smaller effects and to accommodate the complexity of the moderated mediation model, we aimed for a larger sample. This study recruited college students from multiple universities in Fuzhou, Fujian Province, China. Using a stratified random sampling method, questionnaires were distributed to 1,650 students to investigate their current physical exercise participation, sleep quality, level of mobile phone addiction tendency, and psychological resilience. A total of 1,583 valid questionnaires were recovered, yielding an effective response rate of 95.94%. The sample consisted of 708 males and 875 females.

### 2.2 Procedure

This study was conducted in accordance with the principles of the Declaration of Helsinki (44) and was approved by the Scientific Research Ethics Committee of the School of Physical Education and Sports Science, Fujian Normal University (Approval No.: TY2026004), ensuring the protection of participants’ rights and privacy. All participating students signed informed consent forms, and the research purpose, the voluntary nature of participation, and the right to withdraw at any time were explained to the participants in detail. The research team particularly emphasized that all collected data would be kept strictly confidential and that the questionnaires were anonymized to reduce the risk of social desirability bias. Participants’ personal information was properly stored and used solely for research purposes. Throughout the survey process, the researchers ensured that participants were aware that they could withdraw at any time without any negative consequences.

## 3 Measures

### 3.1 Physical exercise scale

This study employed the Physical Activity Rating Scale-3 (PARS-3) developed by Liang (45) to measure physical exercise among college students. The scale consists of three items, measuring exercise intensity, exercise duration, and exercise frequency, respectively, each rated on a 5-point scale. The total score is calculated as: Physical exercise total score = exercise intensity × (exercise duration − 1) × exercise frequency. A score of ≤19 indicates a low level of physical activity, 20–42 indicates a moderate level, and >43 indicates a high level. A higher total score reflects a higher level of physical activity participation.

### 3.2 Sleep quality scale

This study used the Chinese version of the Pittsburgh Sleep Quality Index (PSQI) translated by Liu et al. (46), adapted from the original scale developed by Buysse et al. (47). The scale comprises 24 items in total, including 19 self-rated items and 5 observer-rated items. The scoring includes the 18 self-rated items, covering seven distinct dimensions: subjective sleep quality, sleep latency, sleep duration, sleep efficiency, sleep disturbances, use of sleep medication, and daytime dysfunction. The total score is derived according to the scoring rules for these seven dimensions, with each dimension converted to a score ranging from 0 to 3. Summing the scores of all dimensions yields the PSQI total score, which ranges from 0 to 21. A higher total score indicates poorer sleep quality. According to the scoring criteria of the Pittsburgh Sleep Quality Index, a total score >7 indicates poor sleep quality, a score <4 indicates good sleep quality, and scores falling between these thresholds indicate fair sleep quality. Studies have confirmed that this scale has good reliability and validity among Chinese college students (48).

### 3.3 Mobile phone addiction tendency scale

This study used the Chinese version of the Mobile Phone Addiction Tendency Scale for College Students developed by Xiong et al. (49). The scale consists of 16 items covering four dimensions: withdrawal symptoms, salience behaviors, social comfort, and mood modification. It is a 5-point Likert scale, with responses ranging from “strongly disagree” to “strongly agree” rated from 1 to 5.

### 3.4 Psychological resilience scale

This study used the Chinese version of the Connor–Davidson Resilience Scale (CD-RISC) translated and revised by Yu & Zhang (50) to assess college students’ psychological resilience. The scale consists of three dimensions: tenacity, strength, and optimism, encompassing a total of 25 items, each rated on a 5-point Likert scale ranging from 1 (not true at all) to 5 (true nearly all the time). The total score ranges from 0 to 125, with a higher score indicating better psychological resilience. This scale has good reliability and validity, is widely used to assess individuals’ overall level of psychological resilience in the face of adversity, and has been extensively adopted in measuring psychological resilience among Chinese college students.

The Cronbach’s α coefficients for the PARS-3, PSQI, Mobile Phone Addiction Tendency Scale, and CD-RISC in this study were 0.79, 0.84, 0.83, and 0.91, respectively, indicating acceptable to good internal consistency.

### 3.5 Data analysis

This study used SPSS 27.0, the PROCESS macro (version 4.1) for SPSS, and Microsoft Excel 2010 for data analysis. First, the sample data were preprocessed to test for normality and common method bias. Second, descriptive statistics were calculated by computing the means and standard deviations of each variable. Third, Pearson correlation analysis was conducted among all study variables—physical exercise, mobile phone addiction tendency, and sleep quality—to preliminarily examine the direction and strength of the associations between variables. Fourth, for the mediation analysis, we employed PROCESS Model 4 (51) with physical exercise as the independent variable (X), mobile phone addiction tendency as the mediator (M), and sleep quality as the dependent variable (Y). For the moderated mediation analysis, we employed PROCESS Model 7, which tests whether the X→M path is moderated by psychological resilience (W). The PROCESS syntax used was: Model 4 (X=physical exercise, M=mobile phone addiction tendency, Y=sleep quality) and Model 7 (X=physical exercise, W=psychological resilience, M=mobile phone addiction tendency, Y=sleep quality). Simple slope analysis was used to examine all potentially significant interaction effects. The bootstrap resampling method was applied with 5,000 resamples to calculate 95% bias-corrected confidence intervals (CIs); if the interval did not include zero, the mediation effect was considered statistically significant. Finally, Microsoft Excel 2010 was used for corresponding data statistics, collation, and summarization. All regression models in the mediation and moderated mediation analyses controlled for participants’ sex and grade variables.

## 4 Results and analysis

### 4.1 Common method bias test

Since all data in this study were obtained through self-report from the same participants, common method bias may exist and requires examination. The Harman single-factor test was employed for this analysis (52). The results showed that there were nine factors with eigenvalues greater than 1, and the first factor accounted for 25.131% of the total variance, which is well below the recommended 40% critical threshold. This suggests that no single factor accounted for the majority of the covariance among the measured items, indicating that common method bias was unlikely to be a serious threat to the validity of the study findings. Therefore, it can be concluded that no significant common method bias is present in the study data, thereby enhancing the credibility of the research findings.

### 4.2 Demographic characteristics of the sample

The demographic characteristics of the 1,583 participants are presented in **Table 1**. The sample consisted of 708 males (44.70%) and 875 females (55.30%). In terms of grade distribution, sophomores constituted the largest proportion (n = 497, 31.40%), followed by freshmen (n = 487, 30.80%), juniors (n = 334, 21.10%), and seniors (n = 265, 16.70%).

**Table 1:** Demographic characteristics of the sample (N = 1583).

Category		n	Percentage (%)
Sex	Male	708	44.70
	Female	875	55.30
Grade	Freshman	487	30.80
	Sophomore	497	31.40
	Junior	334	21.10
	Senior	265	16.70

### 4.3 Descriptive statistical analysis of study variables

A descriptive statistical analysis was conducted on the data collected from 1,583 college students regarding physical exercise, sleep quality, mobile phone addiction tendency, and psychological resilience. Physical exercise and sleep quality were represented by total scores, while the remaining variables were represented by item mean scores. While formal normality tests and skewness and kurtosis coefficients (with values of zero indicating a standard normal distribution) are commonly used to assess normality, achieving exact normality is seldom possible in empirical research. Consequently, a distribution is generally considered approximately normal if the data displayed on frequency tables and histograms are roughly symmetrical, or if the skewness and kurtosis values are sufficiently close to zero—criteria that are widely accepted in applied statistics (53). The results are presented in **Table 2**, the skewness and kurtosis values for all variables were close to zero, indicating that the distributions approximated normality and demonstrated relative robustness.

**Table 2:** Descriptive statistical analysis of study variables.

Variable	Min	Max	Mean	SD	Median	Skewness	Kurtosis
Physical exercise	0.00	100.00	27.80	31.17	16.00	1.06	-0.10
Sleep quality	0.00	19.00	8.61	4.44	9.00	-0.59	-0.93
Mobile phone addiction tendency	1.00	5.00	3.25	0.95	3.29	-0.23	-0.65
Psychological resilience	1.00	5.00	3.33	0.89	3.39	-0.26	-0.63

As shown in **Table 2**, the mean total score of physical exercise among college students was 27.80 (SD = 31.17), suggesting that college students’ physical exercise participation was at a moderate level, and that most students possessed adequate exercise awareness and motivation. The mean total score of sleep quality was 8.61 (SD = 4.44), indicating that college students’ sleep quality was below average, with generally poor sleep quality being prevalent. Mobile phone addiction tendency was measured using a 5-point Likert scale, and item mean scores were calculated. The results showed that the mean score of mobile phone addiction tendency was 3.25 (SD = 0.95), which was slightly higher than the theoretical midpoint of 3, indicating that college students’ mobile phone addiction tendency was at a moderate-to-high level. Similarly, psychological resilience was assessed using a 5-point Likert scale, and the item mean score was 3.33 (SD = 0.89), also slightly above the theoretical midpoint of 3, suggesting that college students’ psychological resilience was at a moderate-to-high level. This indicates that college students possess relatively strong resilience and capacity, enabling them to demonstrate perseverance and optimism when facing complex difficulties.

### 4.4 Correlation analysis of variables

In this study, Pearson correlation analysis was used to assess the associations among physical exercise, sleep quality, mobile phone addiction tendency, and psychological resilience (see **Table 3**). The results indicated that physical exercise was negatively correlated with both sleep quality (*r* = −0.42, *p* < 0.001) and mobile phone addiction tendency (*r* = −0.11, *p* < 0.001). That is, higher levels of physical exercise were associated with lower PSQI scores (indicating better sleep quality) and lower levels of mobile phone addiction tendency. Sleep quality was significantly positively correlated with mobile phone addiction tendency (*r* = 0.54, *p* < 0.001), indicating that better sleep quality (lower scores) was associated with lower levels of mobile phone addiction tendency. Among the observed correlations, sleep quality was most strongly associated with mobile phone addiction tendency, while physical exercise demonstrated a moderate correlation with sleep quality. The correlation between physical exercise and mobile phone addiction tendency, though relatively modest in magnitude, was statistically significant.

**Table 3:** Descriptive statistics and interrelations among all of the observed variables.

Control variables	Variables	Physical exercise	Sleep quality	Mobile phone addiction tendency	Psychological resilience
Sex & Grade	Physical exercise	1			
	Sleep quality	-0.42***	1		
	Mobile phone addiction tendency	-0.11***	0.54***	1	
	Psychological resilience	-0.003	0.03	0.04	1

*N* = 1583, ***p < 0.001.

### 4.5 Moderated mediation analysis

To test the proposed hypotheses, we employed the PROCESS macro (version 4.1) for SPSS. Specifically, we first used Model 4 to examine the simple mediating role of mobile phone addiction tendency between physical exercise and sleep quality. Subsequently, we used Model 7 to test the moderated mediation model, in which psychological resilience was specified as a moderator of the relationship between physical exercise and mobile phone addiction tendency (i.e., the first stage of the indirect pathway). Bootstrap resampling with 5,000 resamples was selected, and the significance of the mediation effect was determined by examining whether the 95% confidence interval contained zero; if the confidence interval did not contain zero, the mediation effect was considered statistically significant.

#### 4.5.1 Mediation effect test of mobile phone addiction tendency

**Figure 2:**
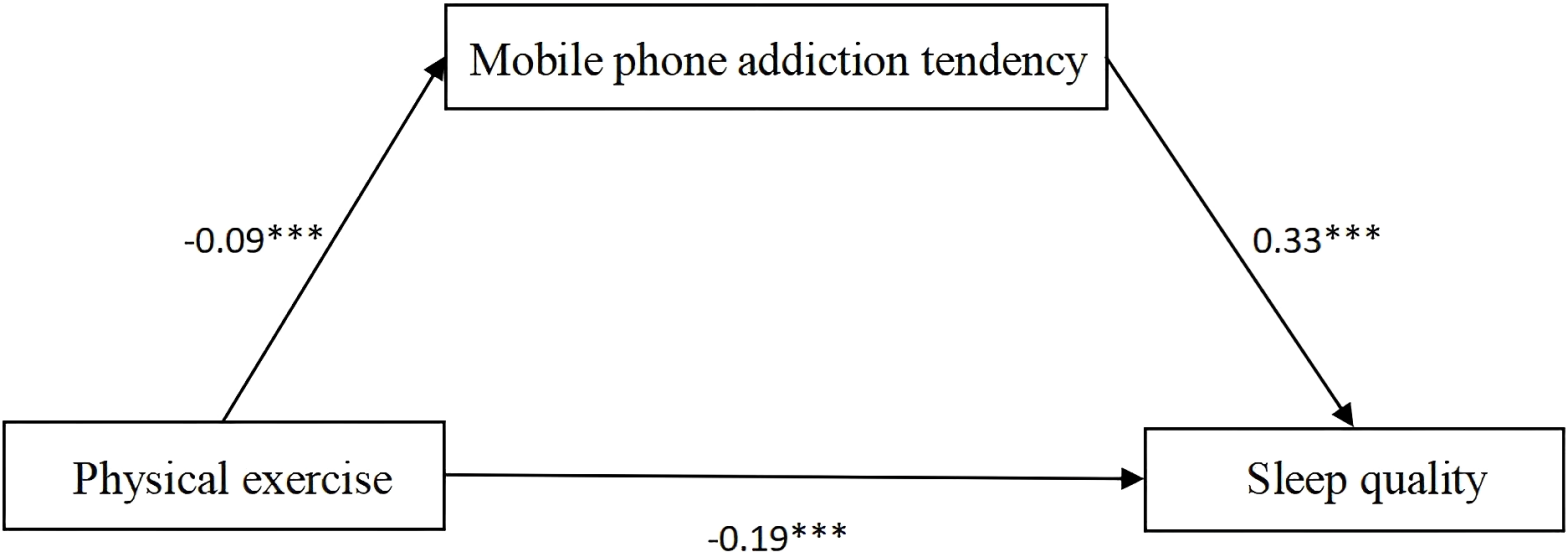
Path coefficients of the mediation model. (***p < 0.001, All coefficients have been standardized).

To explore the pathway underlying the significant association between physical exercise and sleep quality, this study introduced mobile phone addiction tendency as a mediator and used PROCESS Model 4 to test the significance of the mediating role of mobile phone addiction tendency in the relationship between physical exercise and sleep quality. The path coefficients among physical exercise, mobile phone addiction tendency, and sleep quality are presented in **Figure 2**. Physical exercise had a significant negative effect on mobile phone addiction tendency (*β* = −0.09, *p* < 0.001), indicating that higher levels of physical exercise were associated with lower levels of mobile phone addiction tendency. Physical exercise also had a significant negative effect on sleep quality scores (*β* = −0.19, *p* < 0.001), suggesting that higher levels of physical exercise were associated with better sleep quality, reflected in lower PSQI scores. Mobile phone addiction tendency had a significant positive effect on sleep quality (*β* = 0.33, *p* < 0.001), indicating that lower levels of mobile phone addiction tendency were associated with lower sleep quality scores and thus better sleep quality.

As shown in **Table 4**, the direct effect was −0.19 (95% CI [−0.21, −0.17]), accounting for 86.36% of the total effect. The indirect effect was −0.03 (95% CI [−0.04, −0.02]), accounting for 13.64% of the total effect. Specifically, the upper and lower limits of the bootstrap 95% confidence intervals for the effects of physical exercise on sleep quality and for the mediating effect of mobile phone addiction tendency did not contain zero, indicating that physical exercise not only exerts a direct effect on sleep quality but also has an indirect effect on sleep quality through mobile phone addiction tendency. These results provide robust evidence that physical exercise is significantly associated with college students’ sleep quality, and that mobile phone addiction tendency plays a mediating role in this relationship. Thus, Hypothesis 1 and Hypothesis 2 were supported.

**Table 4:** Decomposition of the mediating effect of mobile phone addiction tendency.

	*Effect*	*se*	*t*	*p*	LLCI	ULCI	Effect size
Total effect	-0.22	0.01	-18.43	< 0.001	-0.24	-0.20	
Direct effect	-0.19	0.01	-18.96	< 0.001	-0.21	-0.17	86.36%
Indirect effect	-0.03	0.01	/	/	-0.04	-0.02	13.64%

#### 4.5.2 Moderated mediation effect test of psychological resilience

**Figure 3:**
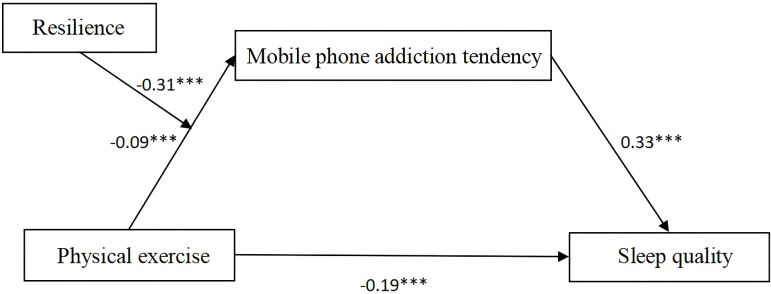
Path coefficients of the moderated mediation model. (***p < 0.001, All coefficients have been standardized).

To test Hypothesis 3, this study employed Model 7 of the PROCESS macro for SPSS to examine the moderating role of psychological resilience, exploring the moderating effect of psychological resilience on the relationship between physical exercise and mobile phone addiction tendency. **Figure 3** shows the path coefficients of the moderated mediation model, illustrating the moderating role of psychological resilience in the relationship between physical exercise and mobile phone addiction tendency. Detailed results of the moderation effect test are presented in **Tables 5** and **6**. As shown in **Table 5**, physical exercise had a significant negative predictive effect on mobile phone addiction tendency (*β* = −0.09, 95% CI [−0.12, −0.05], *p* < 0.001); psychological resilience significantly positively predicted mobile phone addiction tendency (*β* = 0.06, 95% CI [0.01, 0.11], *p* < 0.05). Further analysis revealed that the interaction between psychological resilience and physical exercise had a significant predictive effect on mobile phone addiction tendency (*β* = −0.31, 95% CI [−0.35, −0.27], *p* < 0.001), indicating that psychological resilience moderates the relationship between physical exercise and mobile phone addiction tendency. As shown in **Table 6**, to formally test whether the indirect effect of physical exercise on sleep quality via mobile phone addiction tendency varied as a function of psychological resilience, we computed the index of moderated mediation. The significant index of moderated mediation (Index = −0.10, 95% CI [−0.12, −0.09]). Since this interval did not contain zero, the moderated mediation effect was statistically significant, confirming that psychological resilience significantly moderated the mediating pathway. This result provides statistical confirmation that psychological resilience significantly moderates the mediating pathway, consistent with the interaction effect reported above. These results indicate that the relationship between physical exercise and mobile phone addiction tendency is moderated by psychological resilience. Moreover, neither of the covariates, sex nor grade, reached statistical significance (all *p* > 0.05), indicating that the inclusion of these covariates did not affect the significance of the moderating effect of psychological resilience. Hypothesis 3 was supported.

**Table 5:** Results of the moderation model (outcome variable: mobile phone addiction tendency).

	*Effect*	*se*	*t*	*p*	LLCI	ULCI
physical exercise	-0.09	0.02	-4.83	< 0.001	-0.12	-0.05
psychological resilience	0.06	0.03	2.32	< 0.050	0.01	0.11
Int_1	-0.31	0.02	-15.10	< 0.001	-0.35	-0.27
Sex	-0.02	0.44	-0.54	0.59	-0.11	0.06
Grade	0.02	0.02	0.95	0.34	-0.02	0.06

**Table 6:** Moderation effect analysis at different levels of psychological resilience (Mean ± SD).

Psychological resilience level	*Effect*	*BootSE*	BootLLCI	BootULCI
M-SD (-0.88)	0.18	0.03	0.13	0.23
M (0.00)	-0.09	0.02	-0.12	-0.05
M+SD (0.88)	-0.36	0.03	-0.41	-0.31
Index of moderated mediation	-0.10	0.01	-0.12	-0.09

**Figure 4:**
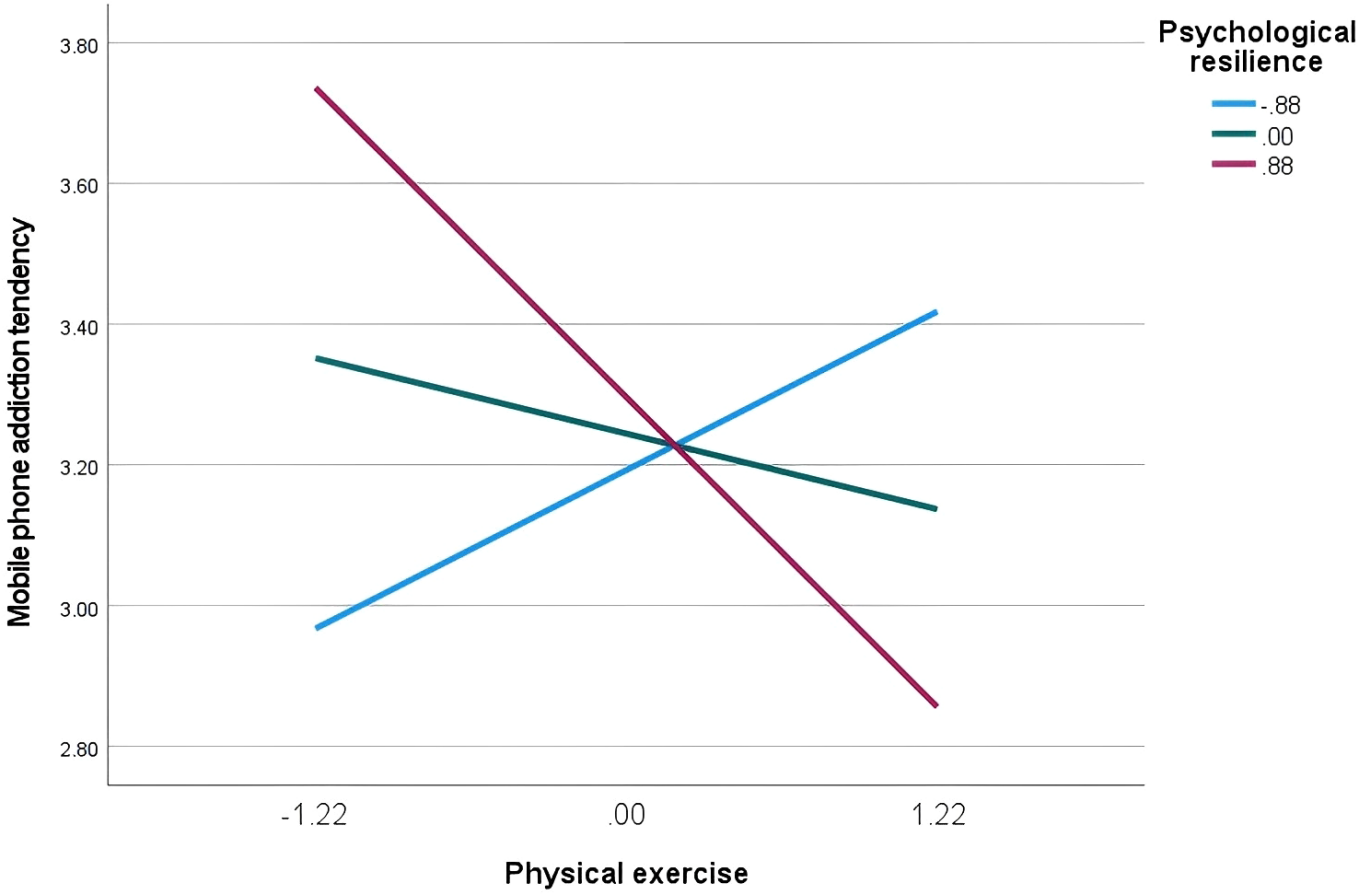
Psychological resilience moderates the relationship between physical exercise and mobile phone addiction tendency. (All coefficients have been standardized).

Furthermore, to examine whether the slopes differed between individuals with high psychological resilience (one standard deviation above the mean) and those with low psychological resilience (one standard deviation below the mean) in the mediation model, we conducted a simple slope analysis to further elucidate the moderation effect and plotted a simple slope diagram (see **Figure 4**). Simple slope analysis revealed that the effect of physical exercise on mobile phone addiction tendency varied significantly across levels of psychological resilience. As shown in **Table 6**, among college students with low psychological resilience (M − 1SD), physical exercise positively predicted mobile phone addiction tendency (*β* = 0.18, 95% CI [0.13, 0.23], *p* < 0.001). For students with moderate psychological resilience (M), physical exercise negatively predicted mobile phone addiction tendency (*β* = −0.09, 95% CI [−0.12, −0.05], *p* < 0.001). Among students with high psychological resilience (M + 1SD), the negative predictive effect of physical exercise on mobile phone addiction tendency was even stronger (*β* = −0.36, 95% CI [−0.41, −0.31], *p* < 0.001). This crossover pattern indicates that psychological resilience not only moderates but also reverses the direction of the association between physical exercise and mobile phone addiction tendency. Specifically, physical exercise appears to be associated with reduced mobile phone addiction tendency only among individuals with moderate to high psychological resilience, whereas among those with low psychological resilience, physical exercise is unexpectedly associated with higher levels of mobile phone addiction tendency.

## 5 Discussion

### 5.1 Physical exercise and sleep quality

This study found that physical exercise was significantly negatively associated with college students’ sleep quality (*β* = −0.19, *p* < 0.001). That is, higher levels of physical exercise were associated with lower PSQI scores, indicating better sleep quality. Hypothesis H1 was thus supported.

The biological basis for the association between physical exercise and sleep quality has been relatively well researched. From the perspective of sleep architecture, laboratory studies have confirmed that acute aerobic exercise can significantly increase both the proportion and intensity of nocturnal slow-wave sleep (SWS). For instance, Aritake-Okada et al. (54) found in a human crossover trial that after participants completed multiple sessions of moderate-intensity aerobic exercise (40% VO_2_max, 40 min per session, 4 times) during the day, nocturnal SWS duration increased by 33% (*p* = 0.005), and slow-wave activity (SWA) was significantly enhanced, accompanied by elevations in core body temperature and the distal-proximal skin temperature gradient. Moreover, further EEG analyses indicated that exercise not only increased SWS quantity but also enhanced SWS stability, reflected in a smoother and more sustained amplitude envelope of delta waves. Park et al. (55), through a rigorous crossover intervention experiment, found that while 60 minutes of vigorous exercise (60% VO_2_max) did not significantly increase total SWS duration, delta power in the N3 sleep stage was significantly enhanced and SWS stability was markedly improved in the early sleep phase. Improvements in SWS signify more efficient clearance of brain metabolic waste and enhanced recovery of synaptic plasticity, serving as core biomarkers of sleep’s restorative function. It can thus be inferred that long-term regular exercise may induce adaptive improvements in the thermoregulatory system, rhythmic enhancement of energy metabolism, and optimization of autonomic nervous system balance, thereby providing a solid physiological foundation for the sleep-promoting effects of exercise. Researchers have explained this from multiple theoretical perspectives, such as energy conservation theory, restorative theory, and brain plasticity theory, suggesting that through increased energy expenditure, promotion of bodily repair demands, and enhancement of neuroplasticity, exercise is associated with deeper and more restorative sleep (56–58).

In addition to its direct biological effects, physical exercise may also be associated with sleep quality indirectly through psychological pathways. Drawing on Bandura’s social cognitive theory as the theoretical framework, this study posits that self-efficacy plays a central role in this process. Social cognitive theory proposes that a “triadic reciprocal determinism” exists among an individual’s behavior, cognition, and environment, in which self-efficacy, as the core cognitive variable, influences beliefs about one’s behavioral capabilities and approaches to coping with environmental challenges (15). For college students, regular physical exercise serves as an effective pathway to enhance self-efficacy; when individuals achieve physical fitness goals through sustained exercise and perceive improvements in bodily functioning, their confidence in their own abilities grows. This enhanced self-efficacy then generalizes to other life domains, including positive beliefs about sleep and improved self-regulation. A large-sample study (N = 10,970) based on the 2024 Chinese College Students’ Physical Activity and Health Follow-up Survey provides direct evidence for this pathway, showing that physical exercise indirectly improves sleep quality through two independent paths—enhanced self-efficacy and emotion regulation—and that these indirect effects dominate the total effect (59). This suggests that beyond merely expending energy and inducing fatigue, exercise, more importantly, operates through a psychological empowerment pathway that enables individuals to believe they can manage their daily routines and regulate their emotions, thereby indirectly optimizing sleep. This aligns closely with the theoretical hypotheses of the present study and lays a logical foundation for the subsequent introduction of the mediating pathway of mobile phone addiction tendency and the moderating process of psychological resilience.

This study indicates that physical exercise is significantly positively associated with sleep quality, a finding that is highly consistent with the conclusions of existing research both domestically and internationally. The meta-analysis by de Almeida et al. (23) demonstrated that physical exercise significantly improves sleep quality and depressive symptoms in both adults and older adults, with aerobic exercise producing the greatest effects. Yang et al. (60), in a systematic review and meta-analysis incorporating 21 randomized controlled trials involving a total of 1,252 college students, found that general aerobic exercise was superior to traditional Chinese exercise in improving college students’ sleep quality, with a statistically significant difference between the two. Further analysis revealed that both general aerobic exercise and traditional Chinese exercise exerted positive improving effects on all seven dimensions: subjective sleep quality, sleep latency, sleep duration, habitual sleep efficiency, sleep disturbances, use of sleep medication, and daytime dysfunction. Furthermore, the meta-analysis by Zhou et al. (61) also indicated that exercise is highly effective in improving both subjective and objective sleep quality. Mind-body exercise, aerobic exercise, and aerobic combined with resistance exercise may be the preferred modalities for improving sleep, with longer exercise periods yielding more pronounced improvements in sleep outcomes, although such improvements may gradually attenuate with increasing age.

### 5.2 The mediating role of mobile phone addiction tendency

This study found that mobile phone addiction tendency played a significant mediating role in the relationship between physical exercise and sleep quality. Specifically, physical exercise was negatively associated with mobile phone addiction tendency (*β* = −0.09, *p* < 0.001), while mobile phone addiction tendency was positively associated with sleep quality scores (*β* = 0.33, *p* < 0.001). The indirect effect was −0.03 (95% CI [−0.04, −0.02]), accounting for 13.64% of the total effect, thus supporting Hypothesis H2. This finding indicates that physical exercise is associated with better sleep quality both directly and indirectly through its association with lower mobile phone addiction tendency, providing empirical evidence for the relationships among physical exercise, sleep quality, and mobile phone addiction tendency.

The mediating role of mobile phone addiction tendency between physical exercise and sleep quality has received increasing empirical support in recent years. Previous research has demonstrated that physical exercise can directly predict college students’ sleep quality and also predict sleep quality through the mediating effect of smartphone use behaviors (62). A meta-analysis conducted by Li et al. (31), encompassing 40 studies with 33,650 college students revealed a significant positive correlation between mobile phone addiction tendency and sleep quality (*r* = 0.28, *p* < 0.001), indicating that the more severe the mobile phone addiction tendency, the poorer the sleep quality, which is consistent with the findings of the present study. Zhang & Liu (63), in a study of 1,905 Chinese college students, found that mobile phone dependence significantly mediated the relationship between physical exercise and sleep quality, with the mediating effect accounting for 27% of the total effect, suggesting that physical exercise not only directly improves sleep quality but also indirectly promotes sleep quality by reducing reliance on mobile phones and prolonging sleep duration. Yin et al. (64) further proposed a serial mediation model of “physical activity, self-control, mobile phone addiction tendency and sleep quality” in a sample of 2,274 college students, finding a standardized regression coefficient of physical activity on mobile phone addiction tendency of *β* = −0.286 and a direct effect on sleep quality of *β* = −0.351, both with *p* < 0.001. Furthermore, Wang (65)also confirmed that students can alleviate mobile phone addiction tendency through sustained healthy physical activity and argued that physical activity affects mobile phone addiction tendency through a chain effect of self-control and resilience. The findings of the present study are consistent with these results, further enriching the theoretical evidence chain that physical exercise influences sleep quality through the pathway of behavioral addiction tendency.

Notably, the mediating effect proportion of mobile phone addiction tendency in this study (13.64%) is smaller than the 27% reported by Zhang & Liu (63) and the 28.6% implied by Yin et al. (64). This discrepancy may stem from several factors. First, differences in sample characteristics—Zhang and Liu’s study included a broader age range of college students, while our sample was limited to universities in a single city—may have contributed to variations in the strength of the associations among variables. Second, differences in measurement instruments for physical activity and mobile phone use may have affected the observed effect sizes. Third, and perhaps most importantly, the relatively modest mediating effect in our study suggests that mobile phone addiction tendency is only one of multiple pathways through which physical exercise may influence sleep quality. Other psychological pathways—such as self-efficacy, emotion regulation, and stress reduction (59, 64, 66) —may also play significant mediating roles, either independently or in combination with mobile phone use behaviors. Future research should examine these multiple pathways simultaneously to provide a more comprehensive understanding of the pathways linking physical exercise to sleep quality.

Smartphone use is characterized by pronounced sedentary behavior. The more time college students invest in their mobile phones, the less time they spend on physical exercise, reflecting a resource competition relationship in which one activity displaces the other. This relationship can be explained from the perspective of behavioral substitution. An experimental intervention study by Precht et al. (67) provided direct evidence for the behavioral substitution pathway: the researchers set up three intervention conditions-reducing smartphone use by 60 minutes per day, increasing physical activity by 30 minutes per day, and implementing both measures simultaneously. The results showed that all three interventions significantly increased weekly physical activity and reduced symptoms of problematic smartphone use, indicating that substituting screen time with exercise is an effective behavioral intervention strategy. Su et al. (34) further found that nighttime exercise can effectively reduce sleep delay caused by pre-sleep smartphone use. Among nighttime exercisers, the frequency and duration of exercise were significantly associated with a lower probability of pre-sleep smartphone use. Pirwani & Szabo (68), in a recently published systematic review, also noted that regular physical activity can reduce problematic mobile phone use among college students. The above studies suggest that the time occupied by exercise would otherwise have been occupied by smartphone use, thus achieving, at the behavioral level, a suppression of mobile phone addiction tendency. From this logic, physical exercise may be associated with lower mobile phone addiction tendency through behavioral substitution, which may attenuate the detrimental effects of mobile phone addiction tendency on sleep.

### 5.3 The moderating role of psychological resilience

This study found that psychological resilience significantly moderated the relationship between physical exercise and mobile phone addiction tendency. Specifically, the interaction term between psychological resilience and physical exercise had a significant negative predictive effect on mobile phone addiction tendency (*β* = −0.31, 95% CI [−0.35, −0.27], *p* < 0.001), indicating that psychological resilience moderates the pathway from physical exercise to mobile phone addiction tendency. The significant index of moderated mediation (Index = −0.10, 95% CI [−0.12, −0.09]) further confirmed that the mediating pathway was conditional on psychological resilience. Notably, neither sex nor grade as covariates reached statistical significance (all *p* > 0.05), suggesting that the observed moderating effect of psychological resilience was robust and independent of these demographic characteristics. Hypothesis H3 was thus supported.

The moderating or mediating role of psychological resilience between physical exercise and behavioral addiction tendency has received increasing empirical attention in recent years, although most studies have tested psychological resilience as a mediator rather than a moderator. The finding in this study that physical exercise is negatively associated with mobile phone addiction tendency among individuals with moderate to high resilience is partially consistent with existing research. Wu et al. (69), in a study of 590 Chinese college students, found that physical activity was significantly negatively correlated with mobile phone addiction tendency behavior (*r* = −0.21, *p* < 0.01), and that psychological resilience and interaction anxiety moderated this relationship. Zhao et al. (66), in a study of college students, also confirmed a significant mediating effect of psychological resilience in the relationship between physical exercise and mobile phone addiction tendency, suggesting that physical exercise can alleviate mobile phone addiction tendency by enhancing psychological resilience. Meanwhile, Wang (65), using a serial mediation model, found that self-control and psychological resilience jointly mediated the relationship between physical activity and mobile phone addiction tendency, with physical activity influencing mobile phone addiction tendency through the serial mediating effect of self-control and psychological resilience. Zeng et al. (70) found that self-control, rumination, psychological distress, and loneliness also exerted mediating and moderating effects between physical exercise and mobile phone addiction tendency. Although the above literature mostly focuses on the mediating role of psychological resilience, mediator and moderator variables are not mutually exclusive in theory. By explicitly specifying psychological resilience as a moderator, this study examined how it changes the strength of the relationship between physical exercise and mobile phone addiction tendency, thus providing a new theoretical perspective for elucidating the role of psychological resilience in this pathway.

Simple slope analysis further revealed a crossover interaction pattern (see **Figure 4**). Among college students with low psychological resilience (M − 1SD), physical exercise positively predicted mobile phone addiction tendency (*β* = 0.18, 95% CI [0.13, 0.23], *p* < 0.001). For students with moderate psychological resilience (M), physical exercise negatively predicted mobile phone addiction tendency (*β* = −0.09, 95% CI [−0.12, −0.05], *p* < 0.001). Among students with high psychological resilience (M + 1SD), the negative predictive effect of physical exercise on mobile phone addiction tendency was even stronger (*β* = −0.36, 95% CI [−0.41, −0.31], *p* < 0.001). This crossover pattern indicates that psychological resilience not only moderates but also reverses the direction of the association between physical exercise and mobile phone addiction tendency. Specifically, physical exercise appears to be associated with reduced mobile phone addiction tendency only among individuals with moderate to high psychological resilience; unexpectedly, among those with low psychological resilience, physical exercise is associated with higher levels of mobile phone addiction tendency.

A plausible explanation for this unexpected finding is that for individuals lacking internal protective resources, exercise participation may inadvertently increase smartphone exposure—for example, through the use of fitness tracking applications, sharing workout data on social media, or listening to music during exercise (34)—thereby exacerbating rather than alleviating problematic phone use. Shang et al. (41), in a large-sample study of over 100,000 Chinese college students, confirmed that smartphone addiction tendency is significantly positively correlated with anxiety and depression, that psychological resilience mediates these relationships, and that higher levels of resilience can mitigate the adverse psychological effects of smartphone addiction tendency. Individuals with high psychological resilience may already employ effective self-regulation strategies to manage their smartphone use, leaving less room for exercise to exert additional inhibitory effects. Conversely, for those with low resilience, exercise may not automatically translate into reduced screen time unless accompanied by explicit behavioral guidance.

### 5.4 Limitations and future research directions

Although this study provides new evidence for the mediating role of mobile phone addiction tendency and the moderating effect of psychological resilience on the relationship between physical exercise and sleep quality among college students, several limitations remain, which should be addressed and expanded upon in future research. First, this study employed a cross-sectional design, with all variables measured at a single time point. Although the moderated mediation model in the PROCESS macro was used to test the indirect and moderating effects among variables, this design cannot inherently establish strict causal relationships. Second, although we controlled for sex and grade in all analyses and reported demographic characteristics, other potential confounding variables—such as socioeconomic status, academic stress, sleep environment, and prior mental health conditions—were not measured. Third, age was not collected in this study, which precludes age-related analyses. Although the sample was drawn from multiple universities in Fuzhou using a stratified random sampling method, which enhanced the diversity of the participant pool, the generalizability of the findings to college students in other regions of China or to non-college populations may be limited. Finally, this study used the PSQI total score as the indicator of sleep quality, but existing meta-analyses indicate that the effect sizes of physical exercise on different dimensions of sleep quality (subjective sleep quality, sleep latency, sleep duration, sleep efficiency, sleep disturbances, and daytime dysfunction) may vary. Similarly, the pathways through which mobile phone addiction tendency impairs different sleep dimensions may also differ.

To address the limitations of the present study and further explore the relationship between physical exercise and college students’ sleep quality, future research may be improved in the following ways. First, future studies should adopt longitudinal tracking designs, measuring physical exercise, mobile phone addiction tendency, and sleep quality at multiple time points, in order to clarify the temporal sequence and causal direction among variables. Second, future research should include a broader set of covariates (e.g., socioeconomic status, academic stress, sleep environment) to further rule out alternative explanations and should collect age data to enable age-stratified analyses. Third, future research should replicate this moderated mediation model using more geographically diverse samples to establish the robustness of these findings across different cultural and regional contexts. Fourth, future studies could analyze each sub-dimension of the PSQI as an outcome variable separately, to identify the influence characteristics of physical exercise and mobile phone addiction tendency on different sleep dimensions, thus providing evidence for developing more targeted sleep intervention strategies. Finally, given the unexpected finding that physical exercise was positively associated with mobile phone addiction tendency among students with low psychological resilience, future research should employ qualitative or mixed-methods approaches to explore the contextual factors that may explain this association.

## 6 Conclusion

The findings of this study indicate that physical exercise is significantly associated with sleep quality among college students, with mobile phone addiction tendency mediating this relationship and psychological resilience moderating—and indeed reversing—the direction of the first-stage pathway. Specifically, physical exercise is directly associated with better sleep quality and is also indirectly associated with better sleep quality through its association with lower mobile phone addiction tendency. Notably, the association between physical exercise and mobile phone addiction tendency depends critically on the level of psychological resilience: among students with high resilience, physical exercise is associated with lower mobile phone addiction tendency; however, among those with low resilience, physical exercise is unexpectedly associated with higher mobile phone addiction tendency. This crossover interaction suggests that psychological resilience may serve as a precondition for the protective benefits of physical exercise on mobile phone use behavior. Overall, despite certain limitations, this study provides important insights into the interplay among physical exercise, sleep quality, mobile phone addiction tendency, and psychological resilience in college students. Moreover, the findings offer valuable references for universities in formulating resilience-informed sleep health promotion strategies for college students, particularly highlighting the need for tailored interventions that address smartphone use behaviors among students with low psychological resilience. Future research should employ longitudinal tracking designs, expand sample sizes, incorporate multi-dimensional psychological variables, and utilize qualitative methods, thereby providing a more solid theoretical and empirical foundation for exercise interventions aimed at promoting sleep health among college students.

## Data Availability

The original contributions presented in the study are included in the article/supplementary material. Further inquiries can be directed to the corresponding author.
